# Supplementary material for: Dominating lengthscales of zebrafish collective behaviour
Source: PLoS Comput Biol. 2022 Jan 13;18(1):e1009394. doi: 10.1371/journal.pcbi.1009394 (PMC8797201; doi:10.1371/journal.pcbi.1009394)
Supplement: S1 Text — The detailed description of the tracking system was introduced in the first section, including the analysis on the overall tracking accuracy. The second section presented the details of the fish age. The third section discussed the details of the analysis, typically the calculation of the correlation functions. The fourth section showed the simulation parameters. The fifth section verified the scaling relationship between persistence length and noise in the Vicsek model. The sixth section presented the comparison between our result and previous results. The last section discussed different ways to collapse the experimental data. (PDF) [file pcbi.1009394.s001.pdf]

SUPPLEMENTARY INFORMATION

Dominating lengthscales of zebrafish collective behaviour

Yushi Yang[1,2*], Francesco Turci[2], Erika Kague[3], Chrissy L. Hammond[3], John Russo[4], C. Patrick Royall[5]

1 Bristol Centre for Functional Nanomaterials, University of Bristol, Bristol, UK
2 H.H. Wills Physics Laboratory, University of Bristol, Bristol, UK
3 Department of Physiology, Pharmacology, and Neuroscience, Medical Sciences, University of Bristol, Bristol, UK
4 Department of Physics, Sapienza Università di Roma, Rome, Italy
5 Gulliver UMR CNRS 7083, ESPCI Paris, Université PSL, Paris, France

* yushi.yang@bristol.ac.uk

1 Details of the Tracking System

1.1 The Apparatus

S1 Fig shows the photo of the apparatus where the large bowl shaped tank is placed inside a paddling pool, overlooked by three cameras. The cameras were triggered by an Arduino microcontroller, to generate synchronised videos using software Pylon Viewer. Our approach is different from a frequently used arrangement in which two orthogonal cameras were placed above and along side a transparent fish tank [1, 2, 3]. This choice is motivated by the relatively large group size (50 fish). To determine 3D trajectories, we need to explicitly consider the optical details such as the distortion of the lens and the refraction of the water [4]. Our setup mitigated the effects of refraction, since the light path from the fish to the camera is only refracted once by the water-air interface.

The entire system were heated by two commercial heaters. The warm water was circulated into the inner bowl with a water pump. The bowl was able to exchange water with outside via small holes drilled inside. The measured temperature in the swimming pool ranges from 23 °C to 26 °C. The water circulation is turned off during observations of fish swimming.

The image size produced by our camera (acA2040 um, Basler) is 2056 pixels × 1540 pixels. We mounted a 6 mm fixed focal length lens (C Series, Edmund Optics) on the camera, which yields a wide view, allowing us to place the camera closer to the fish group. The typical distance between the camera and the fish group is around 2 meters. With such setup, the fish (body length $\sim$ 30 mm) appear as black rods in the videos. S2 Fig shows one typical shape of the fish, and the distribution of the fish sizes.

1.2 Camera Calibration and Water Refraction

The intrinsic parameters, including the camera matrix and the distortion coefficients, were obtained by common camera calibration procedure with the help of a chessboard. The extrinsic parameters of the cameras were determined with a chessboard image floating on the water. By doing so, the origin of the global frame of reference was located at $(x, y, 0)$, and the air-water interface is fixed at $z = 0$. This information is

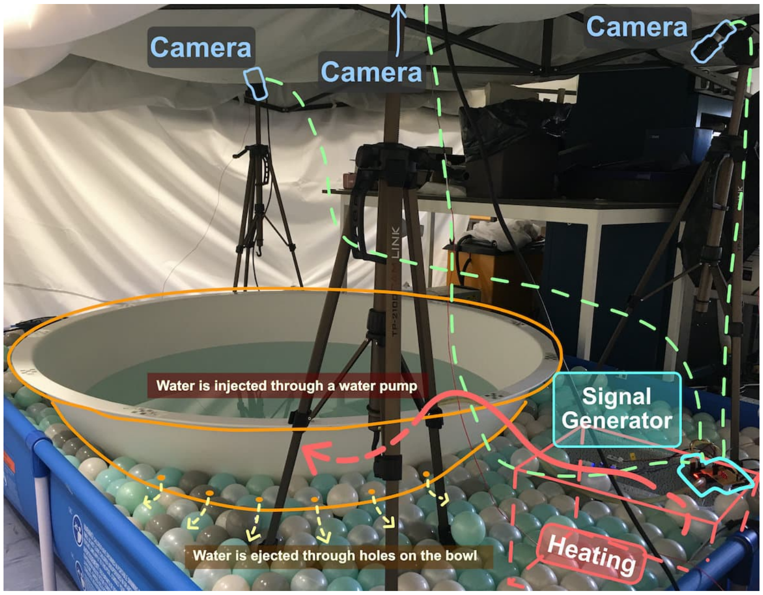

S1 Fig. A photo showing the 3D tracking apparatus featuring the cameras and the bowl–shaped fish tank. The cameras were triggered with signals generated by a Arduino microcontroller.

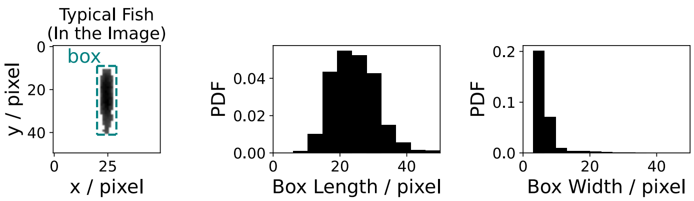

S2 Fig. The shape of fish captured by the cameras. Left: a typical fish shape in the image, and its bounding box. Centre: the distribution of the length of the bonding box. Right: the distribution of the width of the bonding box. The tail of the distribution is because of the bending of the fish body.

used to correct the water-refraction, when we are reconstruct the 3D positions of the fish. S3 Fig (left) shows our method to take account of the air-water interface. With the extrinsic parameters of the cameras, we can calculate the centres of the cameras (in the global frame of reference). Detecting individual fish in the image, we can recast the light path responsible for its formation, visualised as the arrow in the insert of S3 Fig (left). Following the light path, we calculate its intersection with the air-water interface (the plane $z = 0$) and the direction of the light after the refraction following the Snell's law ($n_1 \sin\theta_1 = n_2 \sin\theta_2$). With three cameras, we collect the light from different angles. The intersection of these directions is the location of the fish.

Since our setup is relatively deep (300–400 mm in depth), the effect of the refraction can not be ignored. In S3 Fig (right) we showed the consequence of ignoring the refraction, which is a significant deviation from the true trajectory.

1.3 Tracking Software

The images were loaded using relevant functions from library "opencv", and the fish were separated from the static background by our custom imaging processing script.

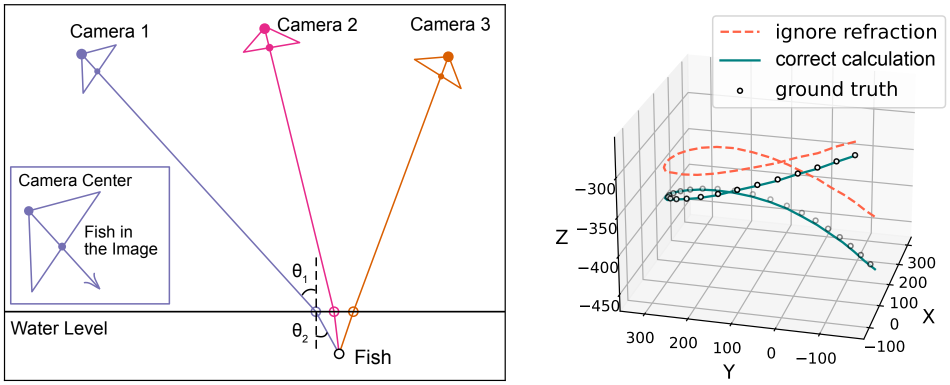

S3 Fig. The air-water interface. Left: the schematic for the calculation of the 3D fish location, with the index change being considered. Right: the effect of the refraction revealed by tracking simulated fish under water. The solid line is the result where the refraction is considered, and the dashed line is the calculation result that ignored the refraction effect. The scatters were ground truth data that were used to generate the simulated image.

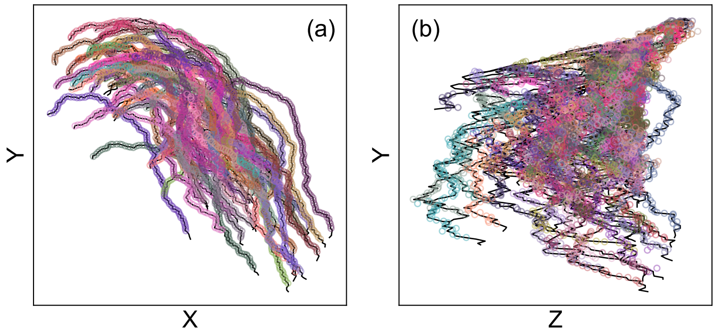

S4 Fig. The tracks of the rendered simulation with the corresponding ground truth. (a) The trajectories projected on XY plane. (b) The trajectories projected on YZ plane. The circles are the tracking results, and the black lines are the ground truth.

The location of fish in different videos were found by our custom 2D tracking code. These 2D locations were then used to calculate the 3D locations of the fish, utilising information about the calibrated camera. Typically, we explicitly considered the refraction of light by the water-air interface, knowing its exact level from the camera calibration procedure. We then link the positions into trajectories using a four-frames predictive procedure [5]. The obtained trajectories were furthered extended into longer ones following Xu's method [6]. All of the aforementioned procedures, including the image processing, 2D feature selection, 3D location and linking, are publicly available in Yushi's GitHub page [7].

1.4 The Accuracy of the Tracking Result

The accuracy of our 3D tracking procedure is evaluated by tracking a rendered fish animation, where the fish were simulated as Vicsek models constrained inside a fish tank. The sizes of the fish and the tank were selected to be close to the experiments.

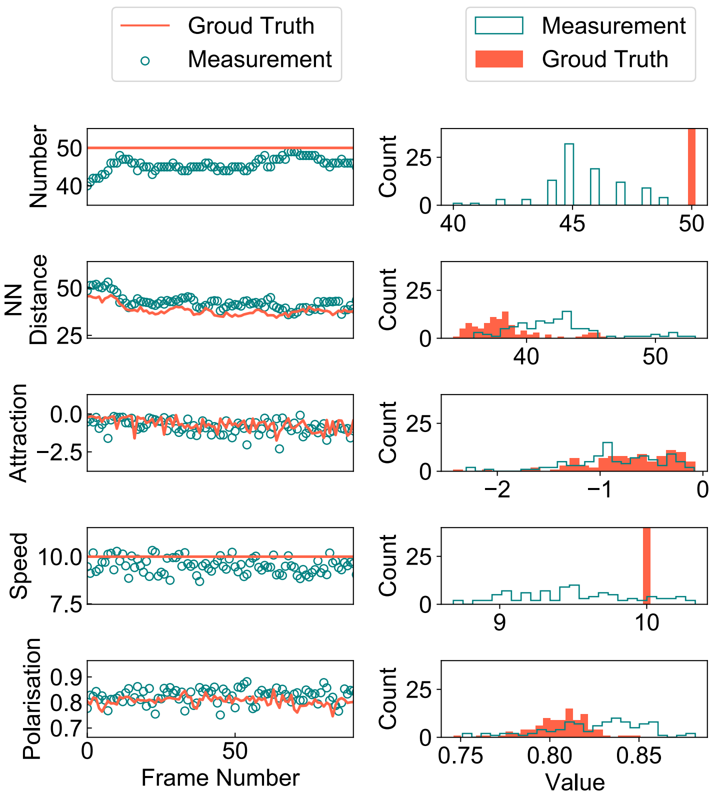

S5 Fig. Comparing the measured behavioural quantities with their corresponding ground truth values. From top to bottom are, the number of tracked fish; the average nearest neighbour distance; the average speed; the polarisation; and the effective attraction. These quantities were calculated frame-by-frame without time averaging. Left: the time evolution. Right: the histogram of different quantities.

We used the "cycles" engine in software "blender" (version 2.91.0 on Ubuntu 20.04) to render the animation, because the ray-tracing renderer can accurately model the refraction of water. The blender model, the rendered movies and the simulation code are available upon request. The rendered simulations are visually similar to the experimental video in terms of the fish density and speed (S4 Video and S5 Video).

The measured trajectories are very close to the ground truth (the simulation result), as illustrated in S4 Fig. The main error from the tracking is the missing of fish from frame to frame. That is, one fish might be tracked in frame i, but it is not tracked in frame $i + 1$. The situation is illustrated in S5 Fig, where only about 90% (≈45) of the fish were tracked on each frame. As a direct consequence, the measured nearest neighbour distance ($\langle l_{\mathrm{nn}} \rangle$) is consistently larger than the ground truth (S5 Fig). The effective attraction, as an alternative quantity which is related to the local density, was found to be more robust, because is it relatively insensitive to any missing coordinates.

2 The Age of the Fish

We determine the age of the fish by measuring their standard body lengths. The results are illustrated in S6 Fig. For group Y1–Y4, we performed the experiment for the same group four times, between August to November in the year of 2019. The fish grew in size during these time as their average body lengths increased from 19.6 mm to 26.5 mm. For group O1–O3, the fish were over 1 years old with the average body length of 30 mm. We performed the experiment for the same group 3 times, in February, 2021. The fish in group O4 were from another different group who are over 1 years old with the average body length of 33.5 mm. The experiments for the O4 group were performed in August, 2019.

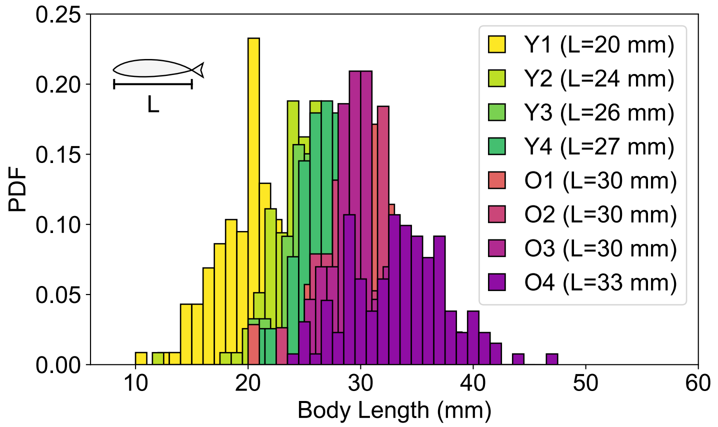

S6 Fig. The distribution of standard body length of different fish groups. The body lengths were measured from the videos manually, with a custom software. The average body length were included in the legends.

3 Details of the Analysis

3.1 Obtaining the positions and velocities

We obtained the positions and velocities of different fish from the linked trajectories. The 3D positions that did not belong to any trajectory were discarded. In our linking process, we allow fish to disappear in some frames, creating voids in the trajectories, which were filled by linear extrapolation.

We segment the fish movement into different sections, of duration 120 seconds, corresponding to a state of the fish group. For each section, we calculated the value of Φ, d_1 and v_0 frame-by-frame, and take their average to characterise the state for this section. For the value of $\langle \epsilon \rangle$ we calculated them from the radial distribution function. For the value of $\langle \tau \rangle$, we collected the trajectories inside the section, and clipped the trajectory if it extended outside the section of interest. Then we calculate the auto-correlation function of the orientations $C_o(t)$ as $C_o(t) = \langle \mathbf{v}_0(i) \cdot \mathbf{v}_0(i+t) \rangle$, where $\mathbf{v}_0 = \mathbf{v}/|\mathbf{v}|$ is the velocity orientation of the fish, and the average is taken over all possible frame numbers (i). The initial decay of $C_o(t)$ is fitted with an exponential decay function $\exp(-t/b)$, by weighting the fitted data by the magnitude of t, and the fitting parameter b is taken as $\langle \tau \rangle$.

3.2 The Auto–correlation Function

We use the auto–correlation function (ACF) to calculate the time–scales of the fish. For scalar variables average over different individuals, such as $\Phi(t)$, $d_1(t)$, and $v_0(t)$, the ACF were calculated as

$$\mathrm{ACF}(\tau) = \langle X(t) X(t+\tau) \rangle,$$

where the bracket $\langle \cdots \rangle$ represents the average over different time points of the entire observation. The result functions were plotted in Fig 2A.

For the orientation ($\mathbf{o}_i = \mathbf{v}_i/|\mathbf{v}_i|$ where $\mathbf{v}$ is the velocity) of fish i, we calculate its ACF as

$$\mathrm{ACF}(\tau)_i = \langle \mathbf{o}_i(t) \cdot \mathbf{o}_i(t+\tau) \rangle_i,$$

where the bracket $\langle \cdots \rangle$ represents the average over different time points along the trajectory of fish i, where the time points fall in one average window of 120 seconds. Then we calculate the average over different fish trajectories, to get one curve plotted in Fig 2B.

3.3 The Radial Distribution Function

We took extra care when calculating the radial radial distribution function ($g(r)$) due to the non-uniform distribution of the fish density, shown in Fig 1C. Specifically, we calculate $g(r)$ as

$$g(r) = \frac{P_{\text{fish}}(r)}{P_{\text{id}}(r)},$$

instead of the counting the number of fish inside a spherical shell. Here $P_{\text{fish}}(r)$ is the probability of two fish having a pair-wise distance of r. The inhomogeneity is handled by biasing the spatial distribution of the ideal gas, *i.e.* independent and random 3D points, to be identical to the spatial distribution of the fish. As a result, the ideal gas will have the same density distribution comparing with the fish, but lack the inherent pairwise structure of the fish. The term $P_{\text{id}}(r)$ is then the probability of a pair of

particles at a separation r for the spatially biased gas. Intuitively, the radio between the two reveals the pair-wise structure of the fish.

4 Details of the Simulation

For the inertial Vicsek model (IVM), we implemented the updating rule for the velocities in the main text,

$$\mathbf{v}_i(t+1) = v_0 \Theta \left[(1-\alpha) \underbrace{v_0 \mathcal{R}_\eta \left[\Theta \left(\sum_{j \in S_i} \mathbf{v}_j(t) \right) \right]}_{\text{Vicsek Model}} + \alpha \mathbf{v}_i(t) \right].$$

The neighbours (S_i) of the ith particle are chosen to be all the particles whose distance is smaller than 1. After the velocities were updated from $\mathbf{v}(t)$ to $\mathbf{v}(t+1)$, we update the positions to be $\mathbf{r}(t+1) = \mathbf{r}(t) + \mathbf{v}(t+1)$. The speed ($v_0$) of the particles was chosen to be 0.1. The number density of our simulation is set to 1 and we implemented a cubic periodic boundary condition. We run the simulation 10^5 time steps before sampling, and we sampled another 10^5 frames to get the results for analysis.

When calculating the orientational relaxation time $\langle \tau \rangle$ for the simulated model, we selected the time when the orientational ACF reaches the value of $1/e$, since the initial ACF can not be easily fitted with an exponential decay. Changing this threshold value will shift the result, but the shift can always be compensated by varying the value of α. All other behavioural quantities were calculated in the same way as the analysis of the experimental data.

5 Numerical Verification of $lp \sim v_0/\eta^2$

For the inertial Vicsek model, we verified the relationship betweel the persistence length (l_p) and noise (η), in the absence of alignment interactions. S7 Fig shows the relationship between the orientational relaxation time ($\tau = l_p/v_0$) of the Vicsek agents and the noise. The relaxation time is obtained by fitting the auto-correlation function of the orientation with an exponential decay. It is clear that the scaling relationship is good, for both the original Vicsek model ($\alpha = 0$) and its inertial counterpart ($\alpha = 0.63$).

6 Comparing with Previous Results

We compared the averaged pairwise distance between the fish with the same results obtained by Miller and Gerlaif [8]. The comparison were presented in S8 Fig, and all the results were close to 20 cm, regardless of the group size.

We also compared the distribution of the polarisation value of our fish (all groups) with the value reported by Miller and Gerlai [9]. The result was plotted in S9 Fig, presenting the distribution of polarisation values for 50 fish. The distribution from our data contains a peak in the disordered region ($\Phi \sim 0.2$) and a tail in the ordered region ($\Phi > 0.5$). Such distribution is very close to the result from Miller and Gerlai.

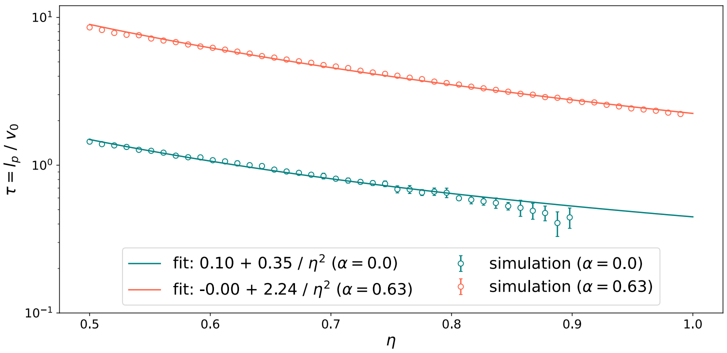

S7 Fig. The relationship between the orientational relaxation time (τ) and the noise value (η) for the agents in the Vicsek model, in the absence of alignment interaction.

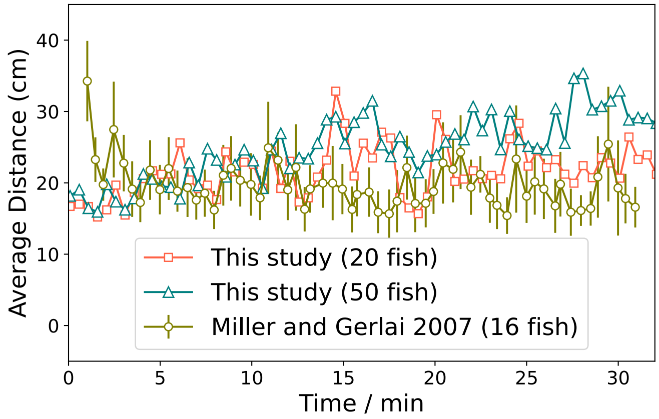

S8 Fig. The average distance values among the fish. Different symbols represent different experiments. For experiments in this study, the fish were young fish (body length < 30 mm), and each scatter represent the average value over 1 second (15 frames). The error bar for Miller and Gerlai's data were from 8 different fish groups.

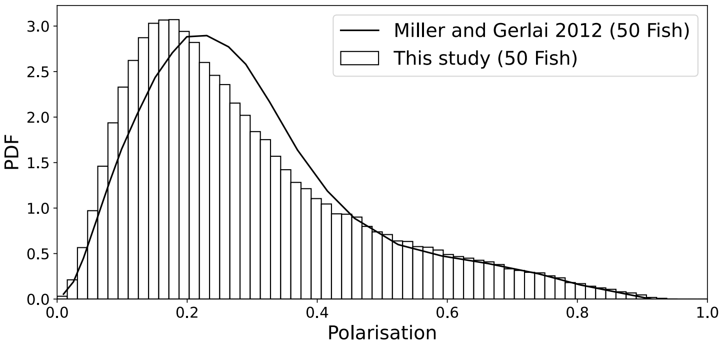

S9 Fig. The distribution of polarisation value of 50 zebrafish. The solid line was obtained from reference [9]. The data from Miller and Gerlai was originally reported as the probability mass function, and we adapted it to the probability density function to compare with our data.

7 Different Variables to Collapse the Data

S10 Fig illustrates four different approaches to collapse the datasets, using the speed, the persistence length, the speed rescaled by the fish body length, and the reduced persistence length κ.

Speed and persistence length alone do not collapse the data. Scaling the speed with the body length or the persistence length with the nearest neighbour distance (as in κ) allows for a much better data collapse. The non-dimensional reduced persistence length (κ), however, allows us for a more insightful interpretation of the data with a direct comparison with the Vicsek model.

In fact, linking the experimental κ values to the one of the Vicesk model allows for a broader interpretation of the results. The observed increase in the polarisation of the fish is a result of the increased κ. In Vicsek's model, where the speed is constant, this is controlled by the noise strength, so that smaller noise leads to longer persistence length and hence polarisation. The noise strength absorbs many potentially complex microscopic mechanisms of decorrelation of the orientation, into a single number.

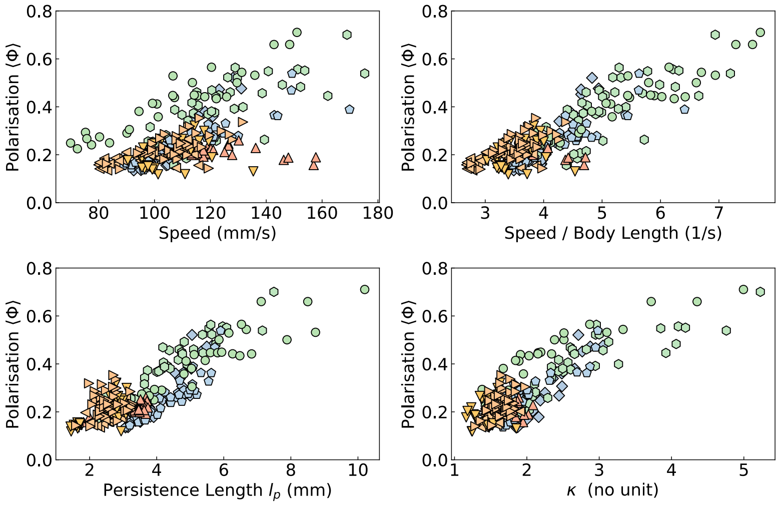

S10 Fig. Different variables used to collapse the data from different groups (Y1-Y4, O1-O4). Different scatters represents different fish groups. The triangles represent the elder fish (O1 - O4) and other symbols represents group Y1 to Y4.

References

1. Cachat J, Stewart A, Utterback E, Hart P, Gaikwad S, Wong K, et al. Three-Dimensional Neurophenotyping of Adult Zebrafish Behavior. PLoS ONE. 2011;6(3):e17597. doi:10.1371/journal.pone.0017597.

2. Mwaffo V, Butail S, Porfiri M. In-Silico Experiments of Zebrafish Behaviour: Modeling Swimming in Three Dimensions. Sci Rep. 2017;7(1):39877. doi:10.1038/srep39877.

3. Rosa LV, Costa FV, Canzian J, Borba JV, Quadros VA, Rosemberg DB. Three- and Bi-Dimensional Analyses of the Shoaling Behavior in Zebrafish: Influence of Modulators of Anxiety-like Responses. Progress in Neuro-Psychopharmacology and Biological Psychiatry. 2020;102:109957. doi:10.1016/j.pnpbp.2020.109957.

4. Parrish JK, editor. Animal Groups in Three Dimensions. Cambridge: Cambridge Univ. Press; 1997.

5. Ouellette NT, Xu H, Bodenschatz E. A Quantitative Study of Three-Dimensional Lagrangian Particle Tracking Algorithms. Exp Fluids. 2006;40(2):301–313. doi:10.1007/s00348-005-0068-7.

6. Xu H. Tracking Lagrangian Trajectories in Position–Velocity Space. Meas Sci Technol. 2008;19(7):075105. doi:10.1088/0957-0233/19/7/075105.

7. Yang Y. Yangyushi/FishPy: First Release; 2020. Zenodo.

8. Miller N, Gerlai R. Quantification of Shoaling Behaviour in Zebrafish (Danio Rerio). Behavioural Brain Research. 2007;184(2):157–166. doi:10.1016/j.bbr.2007.07.007.

9. Miller N, Gerlai R. From Schooling to Shoaling: Patterns of Collective Motion in Zebrafish (Danio Rerio). PLoS ONE. 2012;7(11):e48865. doi:10.1371/journal.pone.0048865.
