# Supplementary material for: Dominating lengthscales of zebrafish collective behaviour
Source: PLoS Comput Biol. 2022 Jan 13;18(1):e1009394. doi: 10.1371/journal.pcbi.1009394 (PMC8797201; doi:10.1371/journal.pcbi.1009394)
Supplement: Attachment — Submitted filename: response_to_reviewers.pdf [file pcbi.1009394.s009.pdf]

Dear Editors,

Thank you for your email of the 25th of October enclosing the two Reviewers' reports. We are grateful for their insightful and positive assessment of our work. In the following response we address the Reviewers' remarks and questions in detail, providing additional information on several aspects of our experimental methods and our analysis.

The main modifications prompted by the Reviewers' reports are:

1. The addition of a new table of variable names for the convenience of the readers.
2. We improved the discussion section, integrating additional references.
3. We added more information regarding the experimental setup, the image analysis and the numerical modelling in the supplementary material.

The specific changes are highlighted in red in one of the attached copies of the revised manuscript.

We hope that the clarifications and improvements included in the revision will allow for its publication on PLOS Computational Biology.

On behalf of all of the authors

Yushi Yang & Francesco Turci

Response to Reviewer #1

We are grateful for the Reviewer's positive assessment of our work and the insightful comments and questions. We address them in detail here below.

> *How does the inaccuracy in tracking (Fig S3), where about 90% of fish are tracked each frame, affect the nearest neighbor statistics? It seems to me that tracking is more likely to lose fish that are occluded or near other fish and this may be a systematic error in the nearest neighbor distance metric. This effect even is shown in the figure. How does this impact the ratio and its correlation to polarization?*

We agree with the Referee that tracking errors leading to missing fish affect our measurements.

To test the robustness of our measurements, we subsampled our data randomly discarding a fraction of tracked fish and recomputing the several collective quantities that we used to characterise the fish behaviour. Figure R1 illustrates the results of this procedure. It is obvious that the nearest neighbour distance (NND) suffered the most from the missing fish, while the polarisation (Φ) and speed were relatively robust. Figure R1 (d) presents the correlation between κ and Φ. Even when we randomly drop 30% tracked fish in all frames, the correlation is still robust, as the polarisation is mainly determined by the speed.

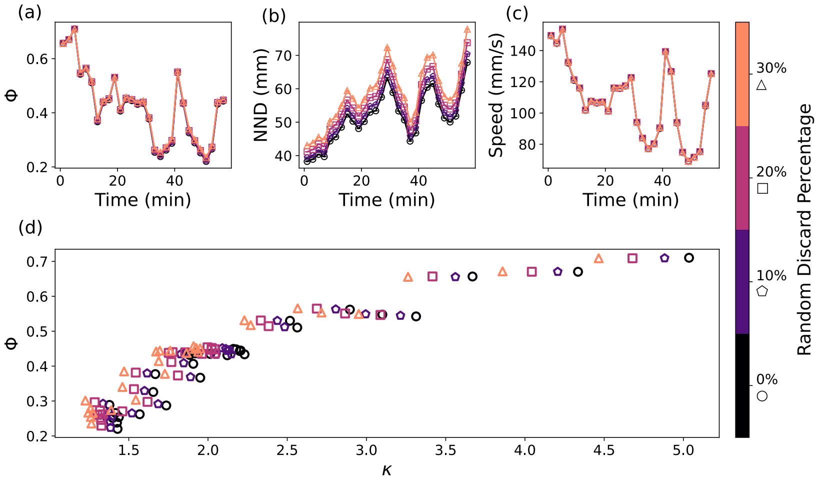

Figure R1: The effect of missing fish on the experimental results from group Y1. (a) The time-evolution of polarisation (Φ). (b) The time-evolution of nearest neighbour distance (NND). (c) The time evolution of averaged individual speed. (d) The correlation between reduced persistence length (κ) and polarisation. The colours of the scatters indicate the number of fish being randomly discarded during the calculation.

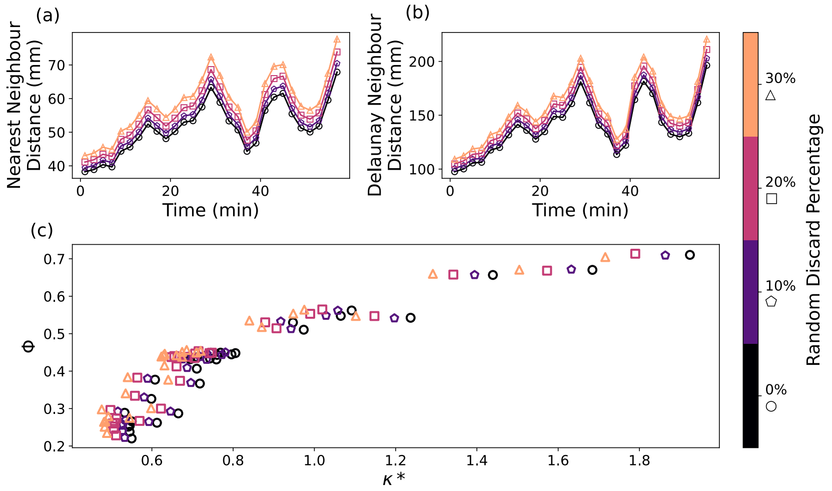

Figure R2: Comparing the nearest neighbour distance and Delaunay neighbour distance, with the experimental results from group Y1. (a) The time-evolution of nearest neighbour distance (NND). (b) The time-evolution of Delaunay neighbour distance. (c) The correlation between the ratio between persistence length and Delaunay neighbour distance, (κ^*) and polarisation. The colours of the scatters indicate the number of fish being randomly discarded during the calculation.

How does the nearest neighbor distance compare with the average distance to neighbors (e.g. through Delauney triangulation)? This may be a more robust statistic.

To address this point, we computed the average neighbour distance (using the Delaunay tessellation to define the local neighbours) and compared it with the nearest neighbour distance, see Fig. R2. We find that the two quantities are very strongly correlated, with the average distance being typically three times larger than the nearest neighbour distance. As suggested by the reviewer, the average over Delaunay neighbours is less sensitive to tracking errors than the nearest neighbour distance. But the difference is modest. If we replace NND with the Delaunay neighbour distance (l_{dn}) in the calculation of κ, writing $\kappa^* = l_p/l_{dn}$, we still get a similar correlation between κ^* and Φ, as shown in Fig. R2 (c). The effect of missing fish is not reduced significantly with the Delaunay neighbour distance.

In the SI, you cite an early literature which remarks on the difficulties of stereomatching through an index change (air-water). Since this is not been done in more recent literature, could the authors provide more details about how the index change affects the measurement of the depth of the fish in the tank?

We took care of the index change by explicitly considering the refraction during the 3D reconstruction of the fish locations.

Our method is summarised in Fig. R3 (left). Our camera calibration yields the extrinsic parameters of the three cameras; hence we know the centre of each camera in the laboratory frame of

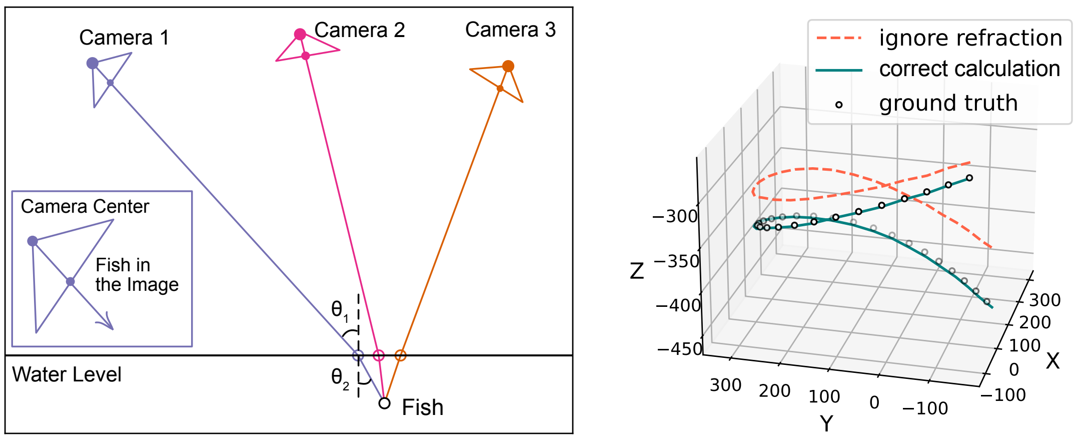

Figure R3: The air-water interface. Left: the schematic for the calculation of the 3D fish location, with the index change being considered. Right: the effect of the refraction revealed by tracking simulated fish under water. The solid line is the result where the refraction is considered, and the dashed line is the calculation result that ignored the refraction effect. The scatters were ground truth data that were used to generate the simulated image.

reference. We placed the calibration board so that the air-water interface is the plane $z = 0$ in the laboratory frame.

With the detected fish location in the image captured by the camera, we can recast the light path responsible for its formation. (The arrow in the insert of the left figure.) From the light path, we calculate its intersection with the air-water interface and the direction of the light after the refraction following the Snell's law ($n_1 \sin\theta_1 = n_2 \sin\theta_2$). With three cameras, we collect the light from different angles. The intersection of these directions is the location of the fish.

Since our setup is relatively deep (300–400 mm in depth), the effect of the refraction can not be ignored. In Fig. R3 (right) we showed the consequence of ignoring the refraction, which is a significant deviation from the true trajectory.

We included the description in the supplementary information (section I.B, "Camera Calibration and Water Refraction").

Fig 2 is very difficult to read as markers are nearly identical to my eye (diamonds and circles) One solution may be replace the diamonds with stars.

We followed the referee's suggestion and provided and a revised version of Fig. 2, in which the diamond markers were replaced with stars.

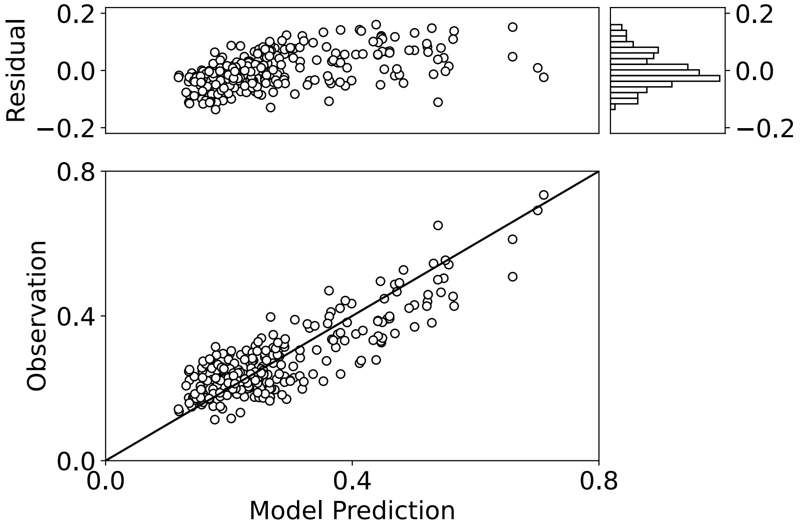

Figure R4: The quality of the linear fitting. The top panel shows the residual, as well as its distribution. The bottom panel shows the comparison between the prediction of the linear model and the observed data.

Also, in Fig 2, there is a large jump in several quantities at 5 min and 40 min. Could these jumps be 'startle' responses? It's been reported that vibrations (from something like a door closing in another room of the building) can cause the fish to respond like this.

We agree with the referee that the rapid responses at min 5 and 40 could be the result of the "startle", but we do not have further data to confirm/reject this possibility.

The laboratory is not fully sound proof. However, we run the experiment so that, after the initial setup, there is no human presence in the experiment room, and we also used an Arduino sound sensor to monitor background noise during the experiment, whose reading is always zero, as the laboratory is relatively quiet.

What are the uncertainties for the fit to the plan for Fig 3b? How "thick" is this plane?

The coefficient of determination $R^2 = 0.73$ in the main text provides an estimate on the uncertainty of the fit for Fig. 3b.

As a further quantification, here we plot our linear model prediction against the observation, illustrating the spread of the residuals across the range. The residuals range between about -0.2 and +0.2. More detailed in formation is available in the supplementary Jupyter notebook (`sCode1.ipynb`).

The cameras are reported as Basler acA2040 um, which from a quick search is 3.2MP. Could this information and the size of fish in px be provided in the SI or text?

The Reviewer has identified the correct information about our camera. We record images with the resolution of (2056 × 1540) pixels. The fish move away and close to the camera, so that their image sizes vary.

At the typical distance (~ 2 meters) between fish and cameras, the image of a single fish (body length ~ 30 mm) is contained in a bounding box of 30×10 pixels, see Fig. R5 (a). The distributions of bounding box sizes are also illustrated in panels (b) and (c). We modified the supplementary information to include these details (Section I.A.).

Activity in fish schools has been shown to depend on temperature. The authors state that the fish are kept in water maintained at 25C. In the paper, this is reported as the living tank temperature. I'll assume that this is the same for the experimental tank. Since this temperature is higher than room temperature, how is the experimental tank maintained at 25C?

The temperature in the experimental tank is similar to the one in the living tanks, in which they are housed for maintenance. The experimental tank is composed of a big bowl, where the fish swims in, and an outer paddling pool. We placed two heaters outside the bowl but inside the paddling pool to maintain the temperature. And we used a water pump to circulate the water, between the (inner) bowl and the (outer) pool. The heating and circulation system is sketched in Fig. R6.

We used commercial heaters with a range from 20 to 30 °C. We keep the heaters constantly at the highest temperature setting, and use a water pump to exchange the heated water with the rest of the tank. We monitor the water temperature in our system with a temperature sensor. The temperature ranged from 23 °C to 26 °C from different measurements. The circulation of the heated water into the bowl was cut off when we observe the fish swimming, in order to avoid the impact of the water flow. (We observed that the fish tend to swim against the flow.) We included these details in the supplementary information.

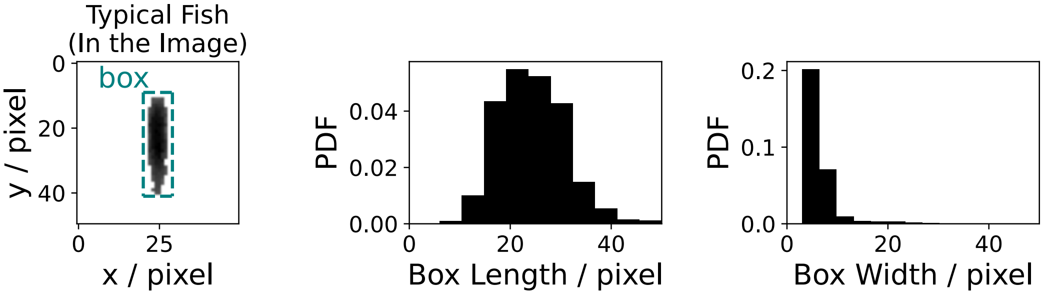

Figure R5: (a) Typical fish image in one of the two cameras, with the corresponding bounding box. (b-c) Size distributions for the length and with of the fish image bounding boxes.

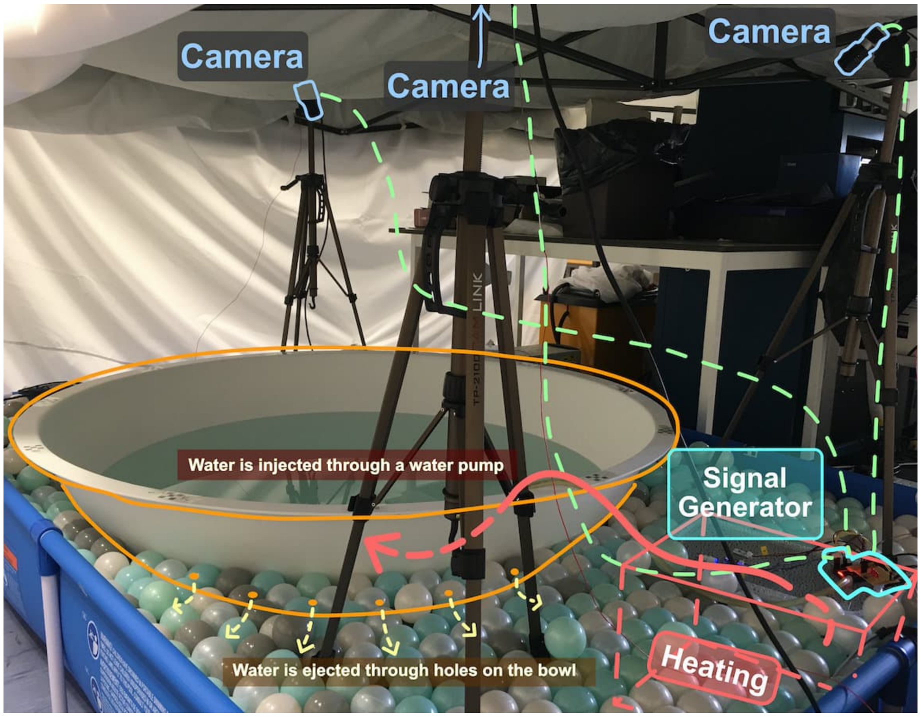

Figure R6: Schematic of the experimental setup overlaid onto a picture of the water tank and experimental instruments.

In the text page 13, the authors report that fish are keep in living tanks with a density of 10 fish/L. But some details are missing. Are the same group of fish used for each test (Y1, Y2, O1, O2, etc)? Or are several living tanks collected to make a school of 50 fish for the experiment? How many fish are there in each living tank?

There are in total 60 young fish living in one tank (12.5 L), and we collect 50 of them randomly every time we perform the experiment. Another 150 older fish were kept in two additional tanks, with about 60 fish in one smaller tank (12.5 L) and about 100 fish living in a larger tank (20 L). For experiments with older fish, we collect 50 of them from one of their living tanks. The selection from the tank was random.

We amend a mistake in the previous version where the fish density was reported to be 10 fish/L instead of 5 fish/L.

Several typos are present in the text an supplemental information. I will include a few here: Text p8 'Tis' → this; SI p 6 'wold' → old.

Thanks to the reviewer comment, we amended our mistakes in the main text.

Response to Reviewer #2

We are grateful for the positive feedback from the Reviewer, as well as the critical comments and questions about our manuscript. We addressed them in detail here below.

> *You put a lot of stress into the fact that the group is studied in 3D and not in 2D. However, Fig 1c suggests that zebrafish are most of the time in a pseudo-2D conformation. Maybe you can report the standard deviation of z and x-y fish locations from the group center of mass?*

In Fig 1c we show that the fish are more likely to be found closer to the walls of the tank than at its centre. This makes the distribution of the fish *not* uniform in 3D. The spread of the distribution (as requested of the referee) informs us of the degree of three dimensional motion of the fish.

In Fig. R7 we show the average coordinates and their standard deviations for the centres of different groups of fish and for an ideal gas (random) distribution of points which we use as a reference to represent the geometry of the tank. The typical z location of the fish group is lower than random, indicating that the fish tend to stay towards the bottom of the tank. This also implies that smaller regions in x, y can be accessed, corresponding to the smaller standard deviations in x, y. The standard deviation in z (however), is similar for the random and the fish groups.

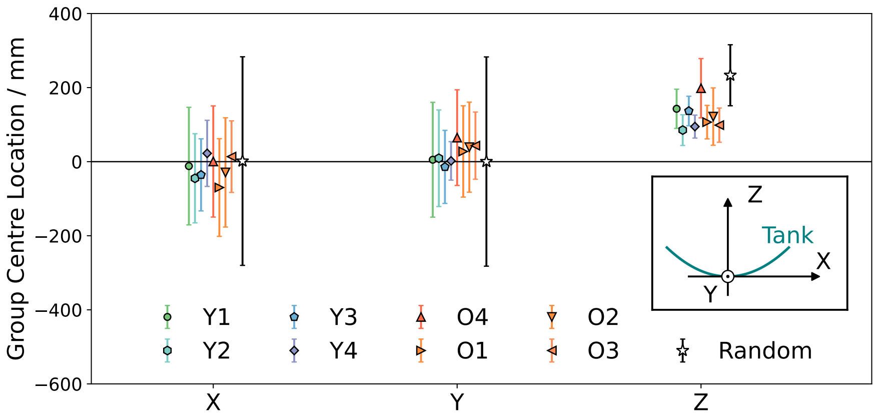

Figure R7: The location of group centres for different fish groups. The y values corresponds to the time-average of the centre locations, and the errorbar indicates the standard deviation. The star markers represents the location of random points uniformly distributed inside the tank.

> *I miss some exploration about how other variables fall into the framework of the two length scales. For example, speed seems to correlate a lot with polarisation (Miller & Gerlai and your figure 2), and it seems to increase alignment (e.g. Heras et al. "Deep attention networks reveal the rules of collective motion in zebrafish", 2019), so I suspect you will see some correlation between speed and the length scales. One of the main contributions of the manuscript is to show the two length scales as useful to describe collective motion, so some effort to try to test whether other variables are useful as well (or more useful) is important.*

In accordance with previous works, the speed of the fish strongly correlates with the polarisation in our experiment, and we re-stressed the fact in our main text. We defined the persistence length as the product between the speed and the orientational relaxation time. This combination allows us to take into consideration changes to the relaxation times that affect the polarisation. We indeed observed some mutant zebrafish to have a larger persistence length *only* because of their larger orientational relaxation time. And this biological difference indeed contributes to the polarisation.

> *One of the main results is the relation between κ and the polarisation. Again, is it much better than the relation between speed (or other variable) and polarisation? This is of utmost important, because the Vicsek model you use to validate your ideas has a constant speed for all agents.*

We appreciate the concern and we present the relationship between polarisation and some other variables in the Fig. R8. In our rationalisation of the experimental results we aimed at data collapse, in order to have a simple, generic relationship explaining the behaviour of different groups of fish in different conditions. (The term "Collapse" means all the experimental points following the same curve.) In Fig. R8 we illustrate four alternative approaches to collapse the datasets, using the speed, the persistence length, the speed rescaled by the fish body length, and the reduced persistence length κ.

Speed and persistence length alone do not collapse the data. Scaling the speed with the body length or the persistence length with the nearest neighbour distance (as in κ) allows for a much better data collapse. The non-dimensional reduced persistence length, however, allows us for a more insightful interpretation of the data with a direct comparison with the Vicsek model.

In fact, linking the experimental κ values to the one of the Vicesk model allows for a broader interpretation of the results. The observed increase in the polarisation of the fish is a result of the increased κ. In Vicsek's model, where the speed is constant, this is controlled by the noise strength, so that smaller noise leads to longer persistence length and hence polarisation. The noise strength absorbs many potentially complex microscopic mechanisms of decorrelation of the orientation, into a single number.

The origin of such decorrelation patterns can be varied: in the present manuscript, we showed that age plays a role; preliminary data that we include in this response show that other more specific factors can be tuned. For example we can study wildtype (wt) and mutant fish with identical age. The mutant fish are designed with genetic modifications affecting their development. In Fig. R9 we show that the relationship between polarisation and reduced persistence length κ still provides a reasonable collapse of the data while the speed/body length scaling does not.

> *Regarding the discussion, stress/relaxation is mentioned in Miller & Gerlai (2012) as a possible explanation of the decrease in polarization of the group after spending some time in the same tank. There is a nice discussion about this effect in Miller & Gerlai, and also some more modern theoretical studies that are worth mentioning (e.g. Perez-Escudero et al. "Adversity magnifies the importance of social information in decision-making", 2017).*

We appreciate the suggestions, and we included these work (Miller & Gerlai 2012, Perez-Escudero et al 2017) into our discussion, as a possible explanation for the change of the local density of the fish.

Minor suggestions:

- *A lot of variables to remember, so I think a table of symbols could be useful.*
- *When possible, I would suggest avoiding the use of several terms for the same thing (confusing to the reader).*
- *Typos and missing GitHub link to code.*

We appreciate these comments and suggestions, and we addressed all of them in the main text.

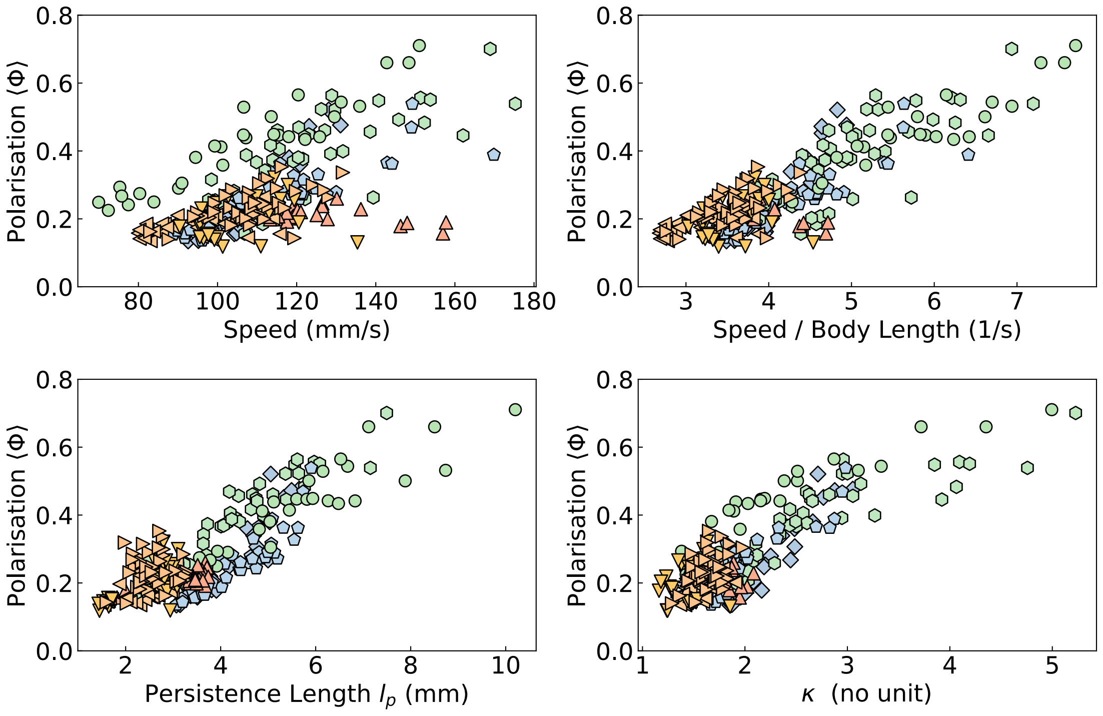

Figure R8: Different variables used to collapse the data from different groups (Y1-Y4, O1-O4). Different scatters represents different fish groups.

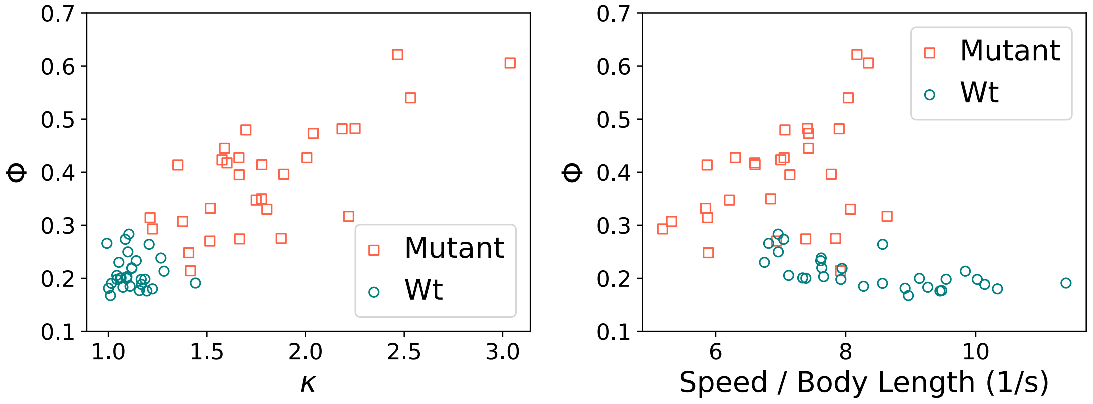

Figure R9: Left: the relationship between polarisation and the reduced persistence length (κ). Right: the relationship between polarisation and the rescaled speed (speed divided by body length).
